# Assembly-free genome comparison based on next-generation sequencing reads and variable length patterns

**DOI:** 10.1186/1471-2105-15-S9-S1

**Published:** 2014-09-10

**Authors:** Matteo Comin, Michele Schimd

**Affiliations:** 1 Department of Information Engineering, University of Padova, Via Gradenigo 6/A, Padova, Italy Full list of author information is available at the end of the article

**Keywords:** alignment-free statistics, next-generation sequencing, pattern discovery

## Abstract

**Background:**

With the advent of Next-Generation Sequencing technologies (NGS), a large amount of short read data has been generated. If a reference genome is not available, the assembly of a template sequence is usually challenging because of repeats and the short length of reads. When NGS reads cannot be mapped onto a reference genome alignment-based methods are not applicable. However it is still possible to study the evolutionary relationship of unassembled genomes based on NGS data.

**Results:**

We present a parameter-free alignment-free method, called $\bar{Under_{2}}$, based on variable-length patterns, for the direct comparison of sets of NGS reads. We define a similarity measure using variable-length patterns, as well as reverses and reverse-complements, along with their statistical and syntactical properties. We evaluate several alignment-free statistics on the comparison of NGS reads coming from simulated and real genomes. In almost all simulations our method $\bar{Under_{2}}$ outperforms all other statistics. The performance gain becomes more evident when real genomes are used.

**Conclusion:**

The new alignment-free statistic is highly successful in discriminating related genomes based on NGS reads data. In almost all experiments, it outperforms traditional alignment-free statistics that are based on fixed length patterns.

## Introduction

The comparison of sequences is fundamental for the analysis of many biological processes. The use of alignment tools like BLAST [1] to assess the degree of similarity between two sequences is a dominant approach. Alignment-based methods produce good results only if the biological sequences under investigation share a reliable alignment. However there are cases where traditional alignment based methods cannot be applied, for example, when the sequences being compared do not share any statistical significant alignment. This is the case when the sequences come from distant related organisms, or they are functionally related but not orthologous. Another drawback is that alignment methods are usually time consuming, thus they cannot be applied to large-scale sequence data produced by NGS technologies.

With the advent of NGS, a large amount of short read data has been generated. These data are used to study many biological problems, such as transcription factor binding sites identification, *de novo *sequencing, alternative splicing, etc. The first step of most studies is to map the reads onto known genomes. However, if a reference genome is not available, the assembly of a template sequence is usually challenging because there may be a large number of repeats within a genome and the short length of reads.

When the NGS reads cannot be mapped onto a reference genome alignment-based methods are not applicable. Moreover the size of NGS data demands the use of very efficient algorithms. For these reasons the comparison of genomes based on the direct comparison of NGS reads has been investigated only recently using alignment-free methods [2].

The use of alignment-free methods for comparing sequences has proved useful in different applications. Some alignment-free measures use the patterns distribution to study evolutionary relationships among different organisms [3-5]. In [6], researchers have shown that the use of *k*-mers frequencies can improve the construction of phylogenetic trees traditionally based on a multiple-sequence alignment, especially for distant related species. The efficiency of alignment-free measures also allows the reconstruction of phylogenies for whole genomes [4,7,8]. Several alignment-free methods have been devised for the detection of enhancers in ChIP-Seq data [9-12] and also of entropic profiles [13,14]. Another application is the classification of protein remotely related, which can be addressed with sophisticated word counting procedures [15,16]. For a comprehensive review of alignment-free measures and applications we refer the reader to [17].

To the best of our knowledge, so far only one group of researchers have compared sets of NGS reads using alignment-free measures based on *k*-mers counting [2]. Here we intend to follow the same approach by adapting our alignment-free pairwise dissimilarity, called *U nder*_2 _[8], for the comparison of two sets of NGS reads. The current study differs from our previous studies [7,8] in the following aspects. First *U nder*_2 _was originally developed to compare pairs of genomic sequences, here we extend it to compare pairs of reads set. Another important aspect is the way patterns are weighted in our similarity score, where we need to consider the expected number of occurrences of a pattern in a set of reads.

Almost all other methods are based on statistics of patterns with a fixed-length *k*, where the performance depends dramatically on the choice of the resolution *k *[4]. Finally, one the most important contributions is the use of reverse and reverse-complement patterns, as well as variable-length patterns to mimic the exchange of genetic material. In summary, in this paper we present a parameter-free alignment-free method, called $\bar{Under_{2}}$, based on variable-length patterns. We will define a similarity measure using variable-length patterns along with their statistical and syntactical properties, so that "uninformative" patterns will be discarded.

The paper is organized as follows. In the next section we review alignment-free methods and their applications. Then we present our contributions, the $\bar{Under_{2}}$ statistic. In the result section we test the performance of several alignment-free measures with both synthetic and real NGS data. In the last section, the conclusions and future work are discussed.

## Previous work

Historically, one of the first papers that introduces an alignment-free method is due to Blaisdell in 1986 [18]. He proposed a statistic called *D*_2_, to study the correlation between two sequences. The *D*_2 _similarity is the correlation between the number of occurrences of all *k*-mers appearing in two sequences. Let *A *and *B *be two sequences from an alphabet Σ. The value *A_w _*is the number of times *w *appears in *A*, with possible overlaps. Then the *D*_2 _statistic is:

$$D_{2}=\underset{w\in \overset{k}{\sum }}{\sum }A_{w}B_{w}.$$

This is the inner product of the word vectors *A_w _*and *B_w _*, each one representing the number of occurrences of words of length *k*, i.e. *k*-mers, in the two sequences. However, it was shown by Lippert *et al*. [19] that the *D*_2 _statistic can be biased by the stochastic noise in each sequence. To address this issue two other popular statistics, called $D_{2}^{S}$ and $D_{2}^{*}$, were introduced respectively in [11] and [20]. This measures were proposed to standardize the *D*_2 _in the following manner. Let *Ã_w _*= *A_w _− *(*n − k *+ 1) *∗ p_w _*and ${\tilde{B}}_{w}=B_{w}-\left(n-k+1\right)*p_{w}$ where *p_w _*is the probability of *w *under the null model and *n *is the length of the strings *A *and *B*. Then $D_{2}^{S}$ and $D_{2}^{*}$ can be defined as follows:

$$D_{2}^{*}=\underset{w\in \overset{k}{\sum }}{\sum }\frac{{\tilde{A}}_{w}{\tilde{B}}_{w}}{\left(n-k+1\right)p_{w}}$$

and,

$$D_{2}^{S}=\underset{w\in \overset{k}{\sum }}{\sum }\frac{{\tilde{A}}_{w}{\tilde{B}}_{w}}{\sqrt{{\tilde{A}}_{w}^{2}+{\tilde{B}}_{w}^{2}}}.$$

These similarity measures respond to the need of normalization of *D*_2_. All these statistics have been studied by Reinert *et al*. [20] and Wan *et al*. [21] for the detection of regulatory sequences. In [2] the authors extend these statistics for genome comparison based on NGS data, and define *d*_2_, $d_{2}^{s}$ and $d_{2}^{*}$. The major difficulties are the random sampling of reads from the genomes and the consideration of double strands of the genome. They tested the performance of *d*2, $d_{2}^{s}$ and $d_{2}^{*}$ on synthetic and real datasets. In particular, the common motif model, introduced by [20], is used to mimic the exchange of genetic material between two genomes, and MetaSim [22] is used to simulate the sequencing. We describe the common motif model in the next sections and propose a more realistic formulation. In this paper we will follow the same experimental setup of [2] and compare our results with these statistics.

## **$\bar{Under_{2}}$**an assembly-free genome comparison based on next-generation sequencing reads and variable length patterns

In this section we describe our parameter-free alignment-free dissimilarity measure, called $\bar{Under_{2}}$, which extends our previous work [8] to the case of NGS reads. The dissimilarity $\bar{Under_{2}}$ is based on two concepts: irredundancy and underlying positioning.

Let's consider two sets of reads *R*_1 _and *R*_2 _that are sampled from two genomes. Every set is composed by *M *reads of length *β *in the alphabet Σ = {*A, C, G, T*}. We say that a pattern in Σ^∗ ^is shared between the two sets of reads if it appears at least once in some read of *R*_1 _and once in some other read of *R*_2_. The notion of irredundancy is meant to remove the redundant patterns, i.e. those patterns that do not convey extra information for the similarity measure. The second driving principle is the fact that, in previous approaches, every position of a read contributes a multiple number of times to the final score.

In the following we address these two issues separately. The goal is to build a similarity measure between the two sets of reads *R*_1 _and *R*_2 _using all exact patterns of any length, Σ^∗^, that are shared between the two sets.

### Removing redundant patterns

One can easily show that most sequences share an unusually large number of common patterns that do not convey extra information about the input. To keep the article self-contained, here we summarize the basic facts already proved in [16] and extend the notion of irredundant common pattern to the case of two sets of reads. If the occurrence of a pattern in a read completely overlaps with the occurrence of another longer pattern, we say that the occurrence of the first pattern is covered by the second one.

**Definition 1 ***(Irredundant/Redundant common patterns) A pattern w is *irredundant *if and only if at least an occurrence of w in R_1 _or R_2 _is not covered by other patterns. A pattern that does not satisfy this condition is called a *redundant common pattern.

We observe again that the set of irredundant common patterns $I_{R_{1},R_{2}}$ is a subset of the well-known linear set of maximal patterns [23]; therefore the number of irredundant common patterns is bounded by *|R*_1_*| *+ *|R*_2_*|*, where *|R*_1_*| *= *|R*_2_*| *= *Mβ*.

A simple algorithm that can discover all such patterns has already been described in [8] and it employs a generalized suffix tree of two sequences. To extend this algorithm to the new input *R*_1 _and *R*_2_, it is sufficient to use the two sets of reads, while maintaining separated the occurrences that belong to the two sets. The construction of the generalized suffix tree and the subsequent extraction of the irredundant common patterns can be completed in time and space linear in the size of sequences [8]. In summary, the notion of irredundancy is useful for removing non-informative patterns, and thus for drastically reducing the number of candidates to be analyzed to estimate the sequence similarity between *R*_1 _and *R*_2_.

### Selecting underlying patterns

The basic idea behind our approach is that a position on the sequences should contribute only once to the final similarity. Traditionally alignment-free statistics fail to comply with this simple rule. In fact, every position, apart from the borders, belongs to *k *different *k*-mers and thus contributes *k *times to the similarity.

In previous works on whole-genome comparison, to solve this problem we used the notions of pattern priority and of underlying pattern [8]. The pattern priority rule is mainly based on the idea of selecting, for each position, those patterns that represent the largest number of matching sites between sequences, and thus that are more likely to be conserved patterns. Here we recall the definition of pattern priority and of underlying pattern from [8], and adapt these concepts to the new settings.

Let's consider the set of irredundant common patterns $I_{R_{1},R_{2}}$ as input. Given two patterns *w *and *w′*, we say that *w *has priority over *w′*, denoted *w → w′*, if and only if either *|w| > |w′|*, or *|w| *= *|w′| *and *w *is less likely to appear in the sequences than *w′*, or *w *and *w′ *have the same length and probability to appear, but the first occurrence of *w *appears before the first occurrence of *w′*. We say that an occurrence *l *of *w *is *tied *to an occurrence *l′ *of another pattern *w′*, if these occurrences (partially) overlap to each other, [*l, l *+ *|w| − *1] *∩ *[*l′, l′ *+ *|w′| − *1]) *≠ *∅, and *w′ → w*. Otherwise, we say that *l *is *untied *from *l′*.

**Definition 2 ***(Underlying patterns) A set of patterns *$U_{R_{1},R_{2}}\subseteq I_{R_{1},R_{2}}$*is said to be the *Underlying set *of {R_1_, R_2_} if and only if:*

*(i) every pattern w in *$U_{R_{1},R_{2}}$, *called underlying pattern, has at least one occurrence in both sets of reads that is untied from all the untied occurrences of other patterns in *$U_{R_{1},R_{2}}\backslash w$, *and*

*(ii) there does not exist a pattern *$w\in I_{R_{1},R_{2}}\backslash U_{R_{1},R_{2}}$*such that w has at least two untied occurrences, one per set of reads, from all the untied occurrences of patterns in *$U_{R_{1},R_{2}}$.

The objective of this definition is to select the most important patterns in $I_{R_{1},R_{2}}$ for each location of the reads in the two sets, according to the pattern priority rule. If a pattern *w *is selected, we filter out all occurrences of patterns with less priority than *w *that lay on the untied locations of *w*, in a simple combinatorial fashion. The complete procedure to discover the set $U_{R_{1},R_{2}}$ can be found in [8]. Here below we give an overview of the algorithm.

***Underlying pattern extraction (Input: R_1_, R_2_; Output: ***$U_{R_{1},R_{2}}$)

*Compute the set of Irredundant common patterns *$I_{R_{1},R_{2}}$.

*Rank all patterns in *$I_{R_{1},R_{2}}$*using the pattern priority rule.*

**for ***Select the top pattern, w, from *$I_{R_{1},R_{2}}$: **do**

  **if ***Check in Γ if w has at least one untied occurrence per sequence that is not covered by some other patterns already in *$U_{R_{1},R_{2}}$**then**

    *Add w to *$U_{R_{1},R_{2}}$*and update the location vector, Γ, in which w appears as untied.*

  **else**

    *Discard w.*

  **end if**

end for

An auxiliary vector Γ, of length *L*, is used to represent all locations of *R*_1 _and *R*_2_. For a pattern *w *in $I_{R_{1},R_{2}}$, we can check whether its occurrences are tied to other patterns by looking at the vector Γ. If some untied occurrences are found, then we can add the new underlying pattern *w *to $U_{R_{1},R_{2}}$, and update the vector Γ accordingly using all the untied occurrences of *w*. In total the extraction of all underlying patterns, using this scheme, takes *O*(*L*^2^) time. A more advanced algorithm with a better complexity, *O*(*L *log *L *log log *L*) time and *O*(*L*) space, can be found in [8].

### Building the $\bar{Under_{2}}$ similarity measure

Our similarity is inspired by the Average Common Subword approach (ACS) [24], where the scores of common patterns found are averaged over the length of sequences. Here we follow the same approach, but, instead of counting all common patterns, we use just the untied occurrences of the underlying patterns, which by definition do not overlap [8]. We can note that the set of underlying patterns $U_{R_{1},R_{2}}$ is not symmetric, in general $U_{R_{1},R_{2}}\neq U_{R_{2},R_{1}}$. Thus, in order to build a symmetric measure, we need to consider both sets.

In ACS the contribution of each position is given by the length of the pattern covering that position. In our approach we use instead the ratio of the number of occurrences for an underlying pattern *w*, and the expected number of occurrences for that pattern. Let's define *occ_w _*as the number of occurrences of *w*, and $untied_{w}^{1}$ as the number of untied occurrences of *w *in *R*_1_. First we compute the score:

$$Score\left(R_{1},R_{2}\right)=\frac{\sum_{w\in U_{R_{1},R_{2}}}|w|*untied_{w}^{1}*\frac{occ_{w}}{E\left[occ_{w}\right]}}{\left|R_{1}\right|}.$$

Recalling that the untied occurrences do not overlap with each other, we notice that the term $|w|*untied_{w}^{1}$ counts the positions where *w *appears without over-lapping any other pattern. For each such position we sum the score $\frac{occ_{w}}{E\left[occ_{w}\right]}$, where *E*[*occ_w_*] is the expected number of occurrences. Note that the expectation of this ratio is exactly 1. This sum is then averaged over the length of the first sequence under examination, *R*_1_. This score is large when the two sequences are similar, therefore we take its inverse. Then, since the total number of occurrences of an underlying pattern *w *present in *R*_1 _is expected to logarithmically increase with the length of *R*_2_, we consider the measure *log*_4_(*|s*2*|*)*/Score*(*s*1*, s*2), where a base-4 logarithm is used to represent the four DNA bases.

To center the formula, such that it goes to zero when *R*_1 _= *R*_2_, we subtract the term log_4 _*|R*_1_*|*. If *R*_1 _= *R*_2 _there will be just one underlying pattern that is equal to the sequence itself. In this case, *Score*(*R*_1_*, R*_1_) will be 1 and the term log_4 _*|R*_1_*| *makes sure that ${\hat{Under}}_{2}\left(R_{1},R_{1}\right)=0$. These observations are implemented in the general formula of ${\hat{Under}}_{2}\left(R_{1},R_{2}\right)$.

$$\begin{array}{c} \;\;\;\;\;\;\;{\hat{Under}}_{2}\left(R_{1},R_{2}\right)=\frac{{\text{log}}_{4}\left|R_{2}\right|}{Score\left(R_{1},R_{2}\right)}-{\text{log}}_{4}\left|R_{1}\right|\\ \bar{Under_{2}}\left(r_{1},R_{2}\right)=\frac{{\hat{Under}}_{2}\left(R_{1},R_{2}\right)+{\hat{Under}}_{2}\left(R_{2},R_{1}\right)}{2} \end{array}$$

Finally, to correct the asymmetry, our similarity measure called $\bar{Under_{2}}$ is the average of the two statistics ${\hat{Under}}_{2}\left(R_{1},R_{2}\right)$ and ${\hat{Under}}_{2}\left(R_{2},R_{1}\right)$.

An important aspect in this formula is the computation of the expected number of occurrences of a pattern *w*. A Markov model usually outperforms the Bernoulli model on biological sequences. In our case the length of reads is relatively short and thus, to avoid overfitting, we will rely on a first order Markov model. In summary, the expectation is computed as *E*[*occ_w _*] = *p_w_M *(*β −|w|*+1), where *pw *is the probability of *w *using the Markov model, *M *is the number of reads and (*β − |w| *+ 1) are the possible occurrences of *w*. Finally, we extend our approach to account for untied occurrences that are present in the reverse, complement, and reverse-complement of each sequence, in order to simulate the DNA strand and the evolution of sequences. For more details about this extension, we refer to [8].

## Experimental results on synthetic and real data

To compare the performance of $\bar{Under_{2}}$ and all *d*-type statistics proposed in [2], we performed several experiments using both simulated and real data.

The common motif model revised

We start from a background sequence which can be either synthetic or a real genomic reference, we call such sequence negative to indicate that no correlation exists between any two of them. For each negative sequence we created a positive one using three different correlation models. The first is the *Common Motif *(*CM *) model introduced in [20]. In the *Common Motif *model a pattern of length five is inserted at position *j *with probability *λ *while the background is left unchanged with probability 1 *− λ*, we chose the same pattern and the same length used in [20,2]. In the *CM *model the pattern inserted is always the same. The second model we adopted is the *Simple Multiple Motifs *(*SMM *), in this model five patterns with length varying from four to six bases are considered. Note that the five patterns are all different now, moreover we consider also their reverse complement in this model. For each position *j *a pattern is inserted with probability *λ*, the pattern to be inserted is chosen so that all five patterns and their reverse complements are inserted with the same probability. The last model introduced is the *Full Multiple Motifs *(*FMM *) model which is a slight variation of *SMM *where, for each pattern, not only the reverse complement is considered, but also the reverse is inserted. The introduction of these two models *SMM *and *FMM *try to mimic the exchange of genetic material between genomes, where regions of variable lengths as well as reverse and reverse complements are important.

### Experimental setup

We test the performance of the different statistics by assessing if sequences from the positive set score higher than those from the negative set. We compute the similarity scores for all pairs of sequences in the positive set and all pairs of sequences in the negative set. Then we sort all scores in one combined list. We consider as positive predictive value (PPV) the percentage of pairs from the positive set that are in the top half of this list, PPV of 1 means perfect separation between positive and negative sequences, while a PPV of 0.5 means no statistical power.

Following the experimental setup of [2], during all the experiments we maintained a constant pattern intensity *λ *= 0.001. For each sequence (either positive or negative) we used MetaSim (http://ab.inf.uni-tuebingen.de/software/metasim/) [22] to generate *M *reads with length *β *= 200 and with standard deviation 0 (*i.e*. all reads have length exactly *β *), in order to obtain an overall coverage *γ *= 5. We will use these parameters for most of the experiments. Except where indicated, exact (*i.e*. no errors) sequencing has been simulated, when errors are considered, the MetaSim preset for 454 model is used with all parameters set to their default values.

For each experimental setup we compute the average score over five runs of $\bar{Under_{2}}$ and of all *d*-type statistics (http://www-rcf.usc.edu/~fsun/Programs/D2_NGS/ D2NGSmain.html). During all simulations, parameters of different algorithms have been maintained fixed, more specifically we used *k *= 5 for *d*-type statistics because this is the best value measured in [2] as well as the best value we observed in a set of preliminary tests.

### Simulations with random background

In this first test we use random sequences as background. Although real datasets are always more desirable than simulations, the use of random sequences is very useful to establish the behavior of alignment-free statistics. Moreover random background sequences can be used to formally prove the statistical power of the *d*-type statistics (see [20,21]).

To simulate data we used the same setup of [2], we considere two different i.i.d. models for negative sequences, uniform background with *p_A _*= *p_C _*= *p_G _*= *p_T _*= 1*/*4 and GC-rich background with *p_A _*= *p_T _*= 1*/*6, *p_C _*= *p_G _*= 1*/*3, we measure the PPV of 40 sequences, 20 positive and 20 negative, as the sequence length *N *varies from 500 to 10000 bases.

**Figure 1:**
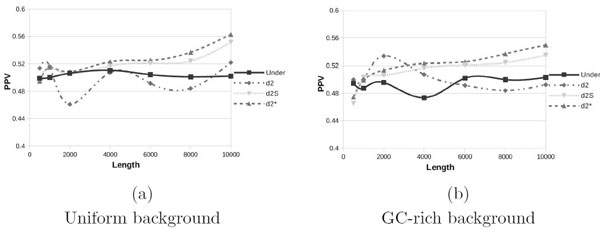
**Positive Predictive Values for uniform and GC-rich background with the Common Motif model**.

Results for the CM model are shown in Figure 1 with both uniform background (a) and GC-rich background (b). Using this setup we observed no significant improvement as *N *grows (recall of 0.5 means no statistical power). All measures are almost aligned around PPV of 0.5 and only for higher values of *N *(4000 or more) $d_{2}^{*}$ and $d_{2}^{s}$ show a slight improvement of their performance. This is explained by the fact that the number of patterns inserted grows with length of the sequence, thus longer sequences from the positive set will have more chance to obtain an higher similarity score. However all methods perform poorly on this dataset, as can be seen from the scale of Figure 1. In general *d*-type statistics need longer sequences or an higher pattern intensity *λ *to improve their predictive power.

**Figure 2:**
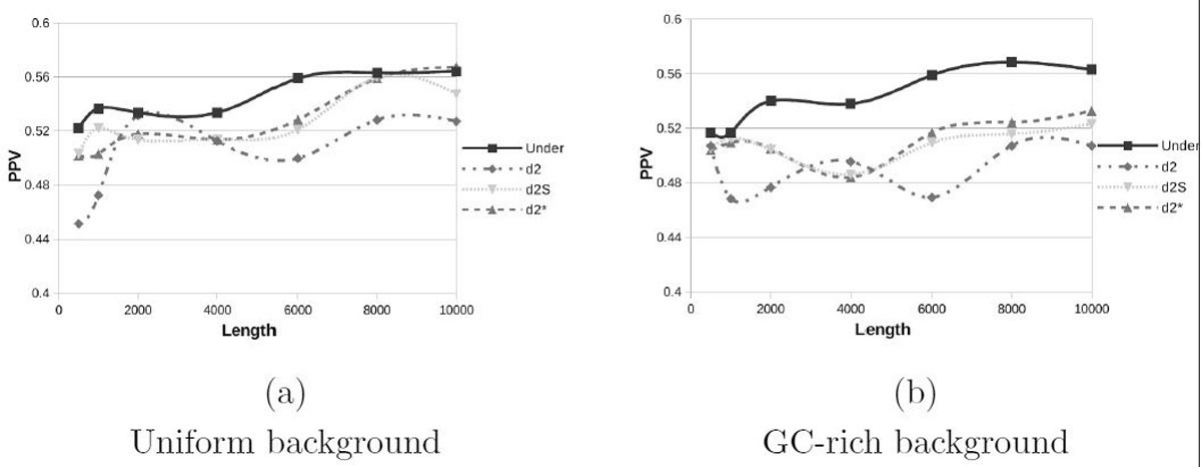
**Positive Predictive Value for uniform and GC-rich background with the Simple Multiple Motifs model**.

**Figure 3:**
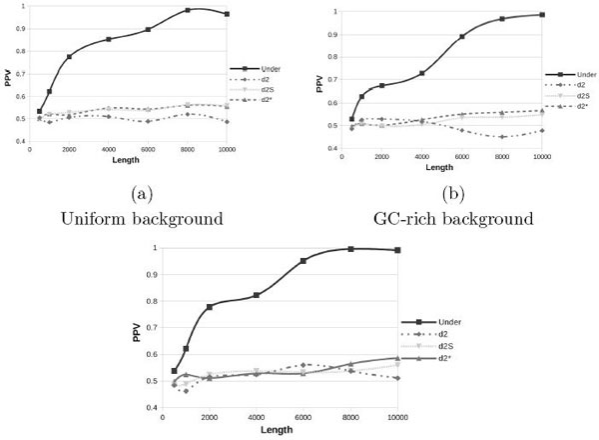
**Positive Predictive Value for uniform and GC-rich background with the Full Multiple Motifs model**.

In Figures 2 and 3 are shown results for the SMM and FMM models, respectively, with uniform (a) and GC-rich (b) backgrounds. The introduction of multiple motifs does not lead to significant performance improvements for *d*-type statistics, even if these statistics consider also the reverse complement. On the other hand we see a slight improvement of $\bar{Under_{2}}$ for the SMM model and a significant improvement for the FMM model, this is due to the fact that introducing the reverse complement (SMM) and also the reverse (FMM) gives better results as the $\bar{Under_{2}}$ statistic explicitly considers them.

By comparing subfigures (a) and (b) of all Figures 1, 2 and 3, we can note that changing the background from uniform to GC-rich produces worse PPV values. However such effect becomes significant only for small values of *N *and when the FMM model is used, while all *d*-type statistics and all other cases of $\bar{Under_{2}}$ are almost immune from such effect, probably because performance in these cases are already poor. Finally in Figure 3(c) we double the coverage, *γ *= 10. If we compare this plot with Figure 3(a) we can note a moderate improvement, especially for longer sequences. Thus, for random backgrounds, increasing the coverage will produce a small performance improvement.

### Simulations with *Drosophila *genome

**Figure 4:**
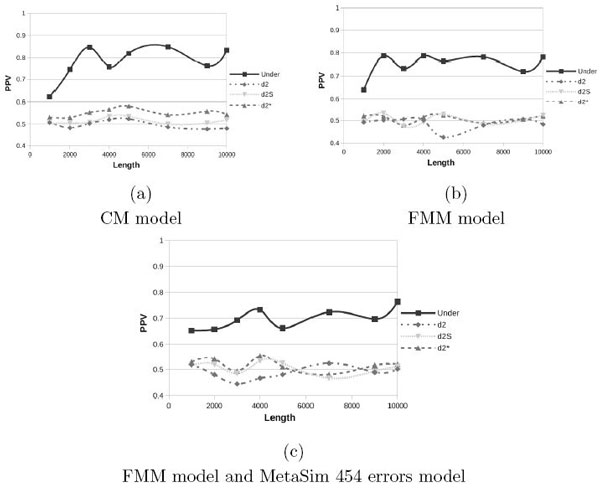
**Positive Predictive Value obtained with *Drosophila *genome as background**.

**Figure 5:**
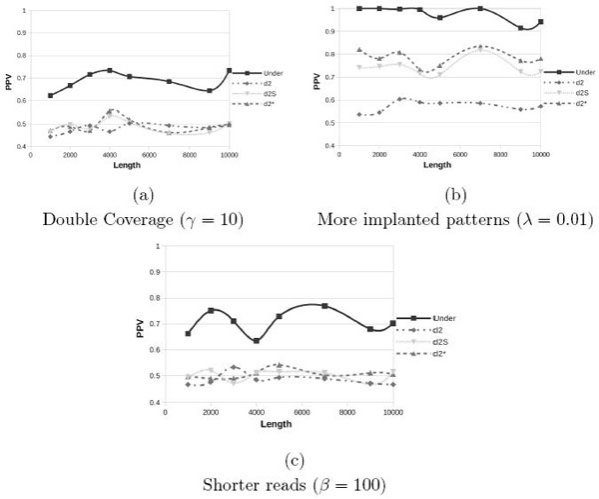
**Positive Predictive Value with *Drosophila *genome as background using the FMM model with 454 sequencing error for various values of parameters (*γ, λ *and *β*)**.

To assess the performance in a more realistic scenario in this section we use as background real genomic sequences from *Drosophila*. We first downloaded all the intergenic sequences of the *Drosophila *genome from FlyBase (http://flybase.org, dmel-all-intergenic-r5.49.fasta) and then we created the negative backgrounds by picking at random 10 sequences for each length varying from 1000 to 10000. We then generated positive sequences using the foreground models CM and FMM described above. To test the impact of sequencing error, we also performed a set of experiments using the 454 error model provided by MetaSim [22] with the FMM foreground, all results are shown in Figure 4 and Figure 5(b).

We observed a consistent trend among all the experiments with $\bar{Under_{2}}$ always outperforming *d*-type statistics. Our measure, in fact, always gives better PPVs at all the tested lengths and for all models. As we introduced sequencing errors results degrade, however this effect is more relevant for short sequences where errors become more important and their effect are, therefore, more visible while at higher lengths the impact of sequencing errors become less significant.

Starting from this latest and more realistic setup, *i.e*. using *Drosophila *genome as background for the FMM model with 454 sequencing errors, we further evaluate how the different parameters affect the performance. Thus we will compare the next plots with Figure 4(c) that has been obtained with the following parameters (*γ *= 5, *λ *= 0.001 and *β *= 200). In Figure 5 we report the PPV values while changing only one parameter at a time. If we double the coverage (*γ *= 10), subfigure (a), the recall values do not improve; only with random backgrounds we see a small improvement (see Figure 3(c)). If we increase the probability to insert a pattern (*λ*) in the FMM model, subfigure (b), as expected, all statistics improve and $\bar{Under_{2}}$ quickly converges to 1. Finally the use of shorter reads (*β *= 100), subfigure (c), does not degrade the recall rates of $\bar{Under_{2}}$ that remains around 0.7.

### Phylogeny of genomes based on NGS data

In this section we test the ability of alignment-free statistics on the reconstruction of whole-genome phylogenies of different organisms. We first selected 12 prokaryotic organisms among the species in [24] for DNA phylogenomic inference. The organisms come from both the major prokaryotic domains: *Archaea*, 6 organisms (Accession No. BA000002, AE000782, AE009439, AE009441, AL096836, AE000520), and *Bacteria*, 6 organisms (Accession No. AE013218, AL111168, AE002160, AM884176, AE016828, L42023). The reference taxonomy is interred using the 16S rDNA sequences and the multiple alignment of these sequences available from the Ribosomal Database Project [25]. Then we perform a maximum likelihood estimation on the aligned set of sequences using Dnaml from PHYLIP [26] in order to compute a reference tree.

We simulate the sequencing process with MetaSim following the same setup as above and then we compute the distance matrices using all statistics. From these distance matrices we derive the taxonomies with the PHYLIP [26] software using neighbor joining (NJ) and the unweighted pair group method with arithmetic mean (UPGMA). We compare the resulting trees with the reference taxonomy using the Robinson and Foulds (R-F) distance. For two unrooted binary trees with *n ≥ *3 leaves, the R-F score is in the range [0, 2*n − *6]. A score equal to 0 means that the two trees are isomorphic, while 2*n − *6 means that all non-trivial bipartitions are different.

The R-F distance between the reference taxonomy and the resulting phylogenetic trees, for all statistics and the two reconstruction methods, are summarized in Table 1. In general $\bar{Under_{2}}$ outperforms all *d*-type statistics obtaining the lower value with both reconstruction methods NJ and UPGMA. We can also observe that $d_{2}^{S}$ and $d_{2}^{*}$ obtain comparable results and, in some cases the former outperforms the latter confirming a similar observation in [2]. This latter experiment confirms that $\bar{Under_{2}}$ is able to detect the genetic signal between unassembled NGS data.

**Table 1:** Comparison of phylogenetic trees of prokaryotic organisms, computed using NGS data, with the reference taxonomy based on the Robinson and Foulds distance.

	$\bar{Under_{2}}$	d_2_	$d_{2}^{S}$	$d_{2}^{*}$
**NJ**	**8**	16	14	14
**UPGMA**	**8**	16	12	14

## Conclusion and future work

In this paper we introduced a parameter-free alignment-free method called $\bar{Under_{2}}$ that is designed around the use of variable-length words combined with specific statistical and syntactical properties. This alignment-free statistic was used to compare sets of NGS reads, in order to detect the evolutionary relationship of unassembled genomes. We evaluate the performance of several alignment-free methods on both synthetic and real data. In almost all simulations our method $\bar{Under_{2}}$ outperforms all other statistics. The performance gain becomes more evident when real genomes are used. As a future direction of investigation, we will try to create a linear time linear space alignment-free measure based also on read quality values.

## Competing interests

The authors declare that they have no competing interests.

## Authors' contributions

M. Comin conceived the study; M. Schimd wrote and tested computer programs for the comparison of reads. All authors drafted and approved the manuscript.

## Declaration

Funding for the publication of this article comes from the Ateneo Project CPDA110239 and the P.R.I.N. Project 20122F87B2 (MI.U.R.).

This article has been published as part of BMC Bioinformatics Volume 15 Supplement 9, 2014: Proceedings of the Fourth Annual RECOMB Satellite Workshop on Massively Parallel Sequencing (RECOMB-Seq 2014). The full contents of the supplement are available online at http://www.biomedcentral.com/bmcbioinformatics/supplements/15/S9.

## References

[B1] Altschul SF Gish W Miller W Myers EW Lipman DJ Basic local alignment search tool Journal of Molecular Biology 1990 215 3 403 410 2231712 10.1016/S0022-2836(05)80360-2

[B2] Song K Ren J Zhai Z Liu X Deng M Sun F Alignment-free sequence comparison based on next-generation sequencing reads Journal of Computational Biology 2013 20 2 64 79 23383994 10.1089/cmb.2012.0228 PMC3581251

[B3] Gao L Qi J Whole genome molecular phylogeny of large dsdna viruses using composition vector method BMC Evolutionary Biology 2007 7 1 1 7 17359548 10.1186/1471-2148-7-41 PMC1839080

[B4] Sims GE Jun SR Wu GA Kim SH Alignment-free genome comparison with feature frequency profiles (ffp) and optimal resolutions Proceedings of the National Academy of Sciences 2009 106 8 2677 2682 10.1073/pnas.0813249106 PMC2634796 19188606

[B5] Qi J Luo H Hao B Cvtree: a phylogenetic tree reconstruction tool based on whole genomes Nucleic Acids Research 2004 32 suppl 2 45 47 15215347 10.1093/nar/gkh362 PMC441500

[B6] Dai Q Wang T Comparison study on k-word statistical measures for protein: From sequence to 'sequence space' BMC Bioinformatics 2008 9 1 1 19 18811946 10.1186/1471-2105-9-394 PMC2571980

[B7] Comin M Verzotto D Whole-genome phylogeny by virtue of unic subwords Database and Expert Systems Applications (DEXA) 2012 23rd International Workshop On, pp. 190-194 (2012)

[B8] Comin M Verzotto D Alignment-free phylogeny of whole genomes using underlying subwords Algorithms for Molecular Biology 2012 7 1 34 23216990 10.1186/1748-7188-7-34 PMC3549825

[B9] Göke J Schulz MH Lasserre J Vingron M Estimation of pairwise sequence similarity of mammalian enhancers with word neighbourhood counts 2012 10.1093/bioinformatics/bts028 PMC3289921 22247280

[B10] Liu X Wan L Li J Reinert G Waterman MS Sun F New powerful statistics for alignment-free sequence comparison under a pattern transfer model Journal of Theoretical Biology 2011 284 1 106 116 21723298 10.1016/j.jtbi.2011.06.020 PMC3146591

[B11] Kantorovitz MR Robinson GE Sinha S A statistical method for alignment-free comparison of regulatory sequences Bioinformatics 2007 23 13 249 255 17646303 10.1093/bioinformatics/btm211

[B12] Comin M Verzotto D Beyond fixed-resolution alignment-free measures for mammalian enhancers sequence comparison 2014 Accepted for Presentation at The Twelfth Asia Pacific Bioinformatics Conference 2014. Proceedings in IEEE/ACM Transactions on Computational Biology and Bioinformatics 10.1109/TCBB.2014.2306830 26356333

[B13] Comin M Antonello M Fast computation of entropic profiles for the detection of conservation in genomes Proceedings of Pattern Recognition in Bioinformatics PRIB, Lecture Notes in BIoinformatics 2013 7986 277 288

[B14] Comin M Antonello M Fast entropic profiler: An information theoretic approach for the discovery of patterns in genomes IEEE/ACM Transactions on Computational Biology and Bioinformatics, 2014 12 10.1109/TCBB.2013.2297924 26356018

[B15] Comin M Verzotto D Classification of protein sequences by means of irredundant patterns BMC Bioinformatics 2010 11 S16 10.1186/1471-2105-11-S1-S16 PMC3009487 20122187

[B16] Comin M Verzotto D The irredundant class method for remote homology detection of protein sequences Journal of Computational Biology 2011 18 12 1819 1829 21548811 10.1089/cmb.2010.0171

[B17] Vinga S Almeida J Alignment-free sequence comparison a review Bioinformatics 2003 19 4 513 523 12611807 10.1093/bioinformatics/btg005

[B18] Blaisdell BE A measure of the similarity of sets of sequences not requiring sequence alignment Proceedings of the National Academy of Sciences 1986 83 14 5155 5159 10.1073/pnas.83.14.5155 PMC323909 3460087

[B19] Lippert RA Huang H Waterman MS Distributional regimes for the number of k-word matches between two random sequences Proceedings of the National Academy of Sciences 2002 99 22 13980 13989 10.1073/pnas.202468099 PMC137823 12374863

[B20] Reinert G Chew D Sun F Waterman MS Alignment-free sequence comparison (i): statistics and power Journal of Computational Biology 2009 16 12 1615 1634 20001252 10.1089/cmb.2009.0198 PMC2818754

[B21] Wan L Reinert G Sun F Waterman MS Alignment-free sequence comparison (ii): theoretical power of comparison statistics Journal of Computational Biology 2010 17 11 1467 1490 20973742 10.1089/cmb.2010.0056 PMC3123933

[B22] Richter DC Felix O F AA Ramona S H HD Metasim--a sequencing simulator for genomics and metagenomics PLoS ONE 2008 3 10 3373 10.1371/journal.pone.0003373 PMC2556396 18841204

[B23] Apostolico A Algorithms and applications 2010 Springer, Berlin, Heidelberg 34-44. Chap. Maximal words in sequence comparisons based on subword composition

[B24] Ulitsky I Burstein D Tuller T Chor B The average common substring approach to phylogenomic reconstruction Journal of Computational Biology 2006 13 2 336 350 16597244 10.1089/cmb.2006.13.336

[B25] Cole J Wang Q Cardenas E Fish J Chai B Farris R Kulam-Syed-Mohideen A McGarrell D Marsh T Garrity G Tiedje J The ribosomal database project: improved alignments and new tools for rrna analysis Nucleic Acids Research 2009 37 141 145 10.1093/nar/gkn879 PMC2686447 19004872

[B26] Felsenstein J PHYLIP (phylogeny inference package), version 3.5 c 1993 Joseph Felsenstein

